# Particular Mal de Meleda Phenotypes in Tunisia and Mutations Founder Effect in the Mediterranean Region

**DOI:** 10.1155/2013/206803

**Published:** 2013-09-04

**Authors:** Mbarka Bchetnia, Nadia Laroussi, Monia Youssef, Cherine Charfeddine, Ahlem Sabrine Ben Brick, Mohamed Samir Boubaker, Mourad Mokni, Sonia Abdelhak, Jameleddine Zili, Rym Benmously

**Affiliations:** ^1^Université de Tunis El Manar, Institut Pasteur de Tunis, Laboratoire de Génomique Biomédicale et Oncogénétique (LR11IPT05), BP74, 13 Place Pasteur, Belvédère, 1002 Tunis, Tunisia; ^2^Hôpital Farhat Hached, Département de Dermatologie, 4000 Sousse, Tunisia; ^3^Hôpital La Rabta, Département de Dermatologie, 1007 Tunis, Tunisia; ^4^Hôpital Habib Thameur, Département de Dermatologie, 1008 Tunis, Tunisia

## Abstract

Mal de Meleda (MDM) is a rare, autosomal recessive form of palmoplantar keratoderma. It is characterized by erythema and hyperkeratosis of the palms and soles that progressively extend to the dorsal surface of the hands and feet. It is caused by mutations in *SLURP-1* gene encoding for secreted mammalian Ly-6/uPAR-related protein 1 (SLURP-1). We performed mutational analysis by direct sequencing of *SLURP-1* gene in order to identify the genetic defect in three unrelated families (families MDM-12, MDM-13, and MDM-14) variably affected with transgressive palmoplantar keratoderma. A spectrum of clinical presentations with variable features has been observed from the pronounced to the transparent hyperkeratosis. We identified the 82delT frame shift mutation in the *SLURP-1* gene in both families MDM-12 and MDM-13 and the missense variation p.Cys99Tyr in family MDM-14. To date, the 82delT variation is the most frequent cause of MDM in the world which is in favour of a recurrent molecular defect. The p.Cys99Tyr variation is only described in Tunisian families making evidence of founder effect mutation of likely Tunisian origin. Our patients presented with very severe to relatively mild phenotypes, including multiple keratolytic pits observed for one patient in the hyperkeratotic area which was not previously reported. The phenotypic variability may reflect the influence of additional factors on disease characteristics. This report further expands the spectrum of clinical phenotypes associated with mutations in *SLURP1* in the Mediterranean population.

## 1. Introduction

In North African populations, genetic transmission indicates a relative abundance of recessive disorders that is clearly associated with consanguineous unions [1]. Mal de Meleda (MDM; OMIM 248300) is a rare autosomal recessive skin disease with a prevalence in the general population estimated to be 1 in 100 000. Meleda refers to an Adriatic island of the Dalmatia region of Croatia, where the occurrence of the disease as an entity of its own among the various forms of Keratosis palmoplantaris hereditaria was first described [2]. The largest MDM series were reported from North Africa: Tunisia (11 families with 45 patients) and Algeria (16 families with 21 patients) [3, 4]. MDM is characterized by erythema and hyperkeratosis of the palms and soles, extending to the dorsal aspects of the hands and feet (known as transgrediens) and perioral erythema and psoriasiform plaques on the elbows and knees. At the histological level, hyperkeratosis, hypergranulosis, and acanthosis are observed [5]. There are no specific therapies to correct the underlying genetic defect due to mutations in *SLURP-1* gene located on chromosome 8q24.3. Until now, the most significant molecular advance of this disease has been the identification of causative mutations and the analysis of the biological role of SLURP-1 protein in the epidermis [6].

The results of previous laboratory studies showed the distribution of only four MDM mutations in North Africa p.Cys99Tyr (5 families), p.Cys77Ala (2 families), p.Gly86Arg (1 family), and 82delT (17 families). The mutations previously reported in Tunisian families were p.Cys99Tyr, p.Cys77Ala, and 82delT.

Herein, we further describe three new Tunisian MDM families with the mutations 82delT and p.Cys99Tyr and presenting higher phenotypic variability.

## 2. Materials and Methods

### 2.1. Subjects

Three unrelated pedigrees of Tunisian descent with MDM phenotype (families MDM-12, MDM-13, and MDM-14) were referred to our laboratory to confirm diagnosis of MDM phenotype. In families MDM-12 and MDM-13, there was only one affected individual (aged of 40 years). In family MDM-14, there was 2 affected siblings that aged 2 and 13 years, respectively. The family history disclosed that the parents in families MDM-12 and MDM-14 are double and first cousins, respectively, whereas in family MDM-13, no consanguineous relationship between the parents was known. In all families, the parents were endogamous without any skin symptoms and MDM was inherited in an autosomal recessive manner (Table 1). Ethical guidelines were followed, and informed consent was obtained from all participants for the genetic investigation.

### 2.2. Mutational Analysis

Blood samples were drawn from each participant family member. DNA extraction from peripheral blood leucocytes was performed using standard procedures. Mutation analysis was carried out in the affected members. All exons of the *SLURP-1* gene with adjacent sequences of exon-intron borders were amplified by PCR with primers and conditions described previously [4]. Mutation screening was performed by direct sequencing using Big Dye terminator technology (ABI 3130), and sequences were analyzed using Bioedit packages.

### 2.3. Generation of SLURP-1 Molecular Models

A model of the three-dimensional structure of SLURP-1 protein wild type and with p.Cys99Tyr variation was realized in order to assess the potential effect of p.Cys99Tyr mutation on the 3D conformation of SLURP-1 protein. *In silico*, modeling was performed by using I-Tasser online server [7] and PyMOL viewer [8].

## 3. Results and Discussion

### 3.1. Results

#### 3.1.1. Clinical Findings

**Figure 1:**
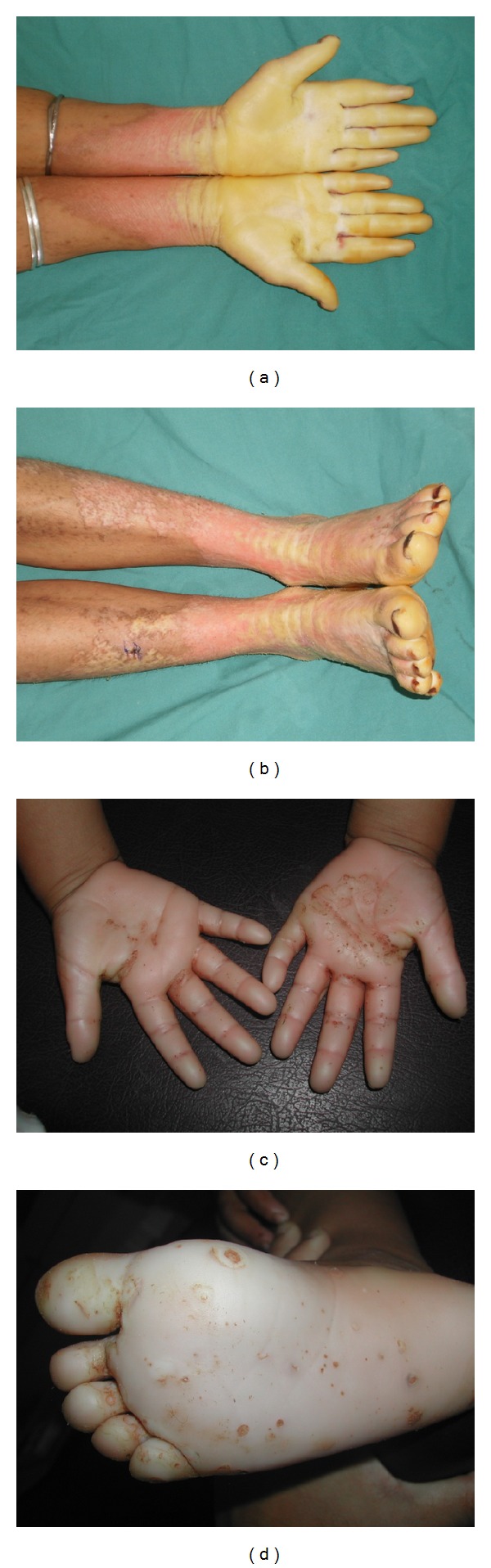
Clinical manifestations of MDM. Pronounced yellowish erythematous transgrediens palmoplantar keratoderma over the hands and feet of patient belonging to family MDM-12 ((a)-(b)). Transparent hyperkeratosis with red border delimiting the hyperkeratotic area and multiple keratolytic pits in one patient belonging to family MDM-14 ((c)-(d)).

In family MDM-12, the patient is the offspring of parents with double consanguinity. She exhibited abnormal keratinization and yellowish erythematous lesions. The age of onset was during the first months of life. Yellow keratoderma of the palms and soles was outlined by a red scaly border in a “glove-and-socks” distribution. Hyperkeratosis spreads from the palms and soles to other parts of the body, such as dorsal aspects of the hands and feet, elbows, knees, perioral regions, and lower legs. Conical distal phalanges and nail changes including thickening of the nails were also noted. This phenotype resulted in severe functional restriction of the hands and feet (Figures 1(a) and 1(b)). In this family, two nonaffected members presented visual impairment problems. The patient in family MDM-13 presented the main clinical features of MDM without other types of complications. In both families, MDM-12 and MDM-13, there was no improvement of the hyperkeratosis with age.

In family MDM-14, the two affected siblings were still young (2 and 13 years). They presented with a mild MDM phenotype characterized by slightly erythematous keratotic plaques that were light red in color and not perfectly apparent. Multiple small keratolytic pits were observed over the palmoplantar surface (Figures 1(c) and 1(d)). They consist of 2–5 mm pits outlined by brownish red erythema. They were variably distributed on the hyperkeratosis area and more frequently on the plantar surface. The rest of the skin was normal.

Table 2 summarizes the clinical characteristics of all the studied patients.

#### 3.1.2. Mutational Analysis

In families MDM-12 and MDM-13, sequencing of PCR-amplified *SLURP1* gene revealed the existence of a nucleotide deletion, 82delT at homozygous state in the affected individuals. This frame shift variation is leading to a creation of a premature stop codon at amino acid position 32 within exon 2 and to the synthesis of a truncated protein.

In family MDM-14, we identified a missense mutation which changes G to A at nucleotide position 297 within exon 3. It leads to an amino acid change from cysteine to tyrosine at codon 99 (p.Cys99Tyr) (Table 1). 

#### 3.1.3. Comparative Modeling of SLURP-1 Three-Dimensional Structures

**Figure 2:**
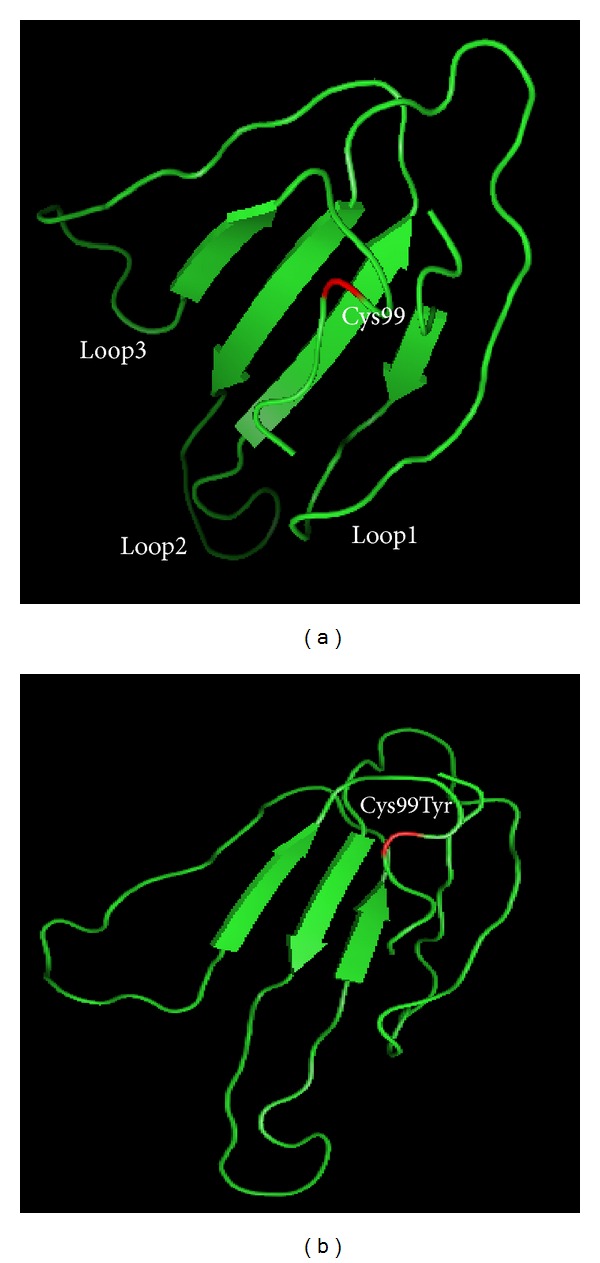
In silico modeling of the SLURP1 wild type (a) and SLURP1 with the p.Cys99Tyr mutation (b). The model shows the possible structural differences between the mutant and wild-type proteins. The presence of the p.Cys99Tyr variation led to a new protein folding missing the loops conformation. Structures are determined from amino acid sequences with I-Tasser online server and figures produced with PyMOL viewer. The p.Cys99Tyr variation position is showed by red.

Comparative modeling of SLURP-1 wild type and SLURP-1 with p.Cys99Tyr variation showed difference in the protein 3D conformation. The p.Cys99Tyr probably destabilizes the whole structure and particularly the third loop of the three-finger fold by changing the folding properties (Figure 2).

### 3.2. Discussion

**Figure 3:**
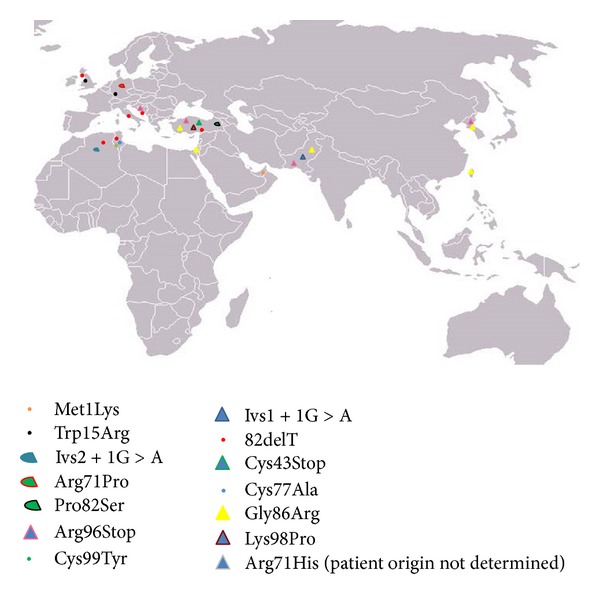
Geographic distribution of the *SLURP-1* gene reported mutations. Met1Lys (Emirates Bedouin [15]); Ivs1 + 1G > A (Pakistani [16]); Trp15Arg (German [15], Scottish [3], and Dutch [17]); 82delT (Tunisian [3], Scottish [3], Algerian [3], Croatian [9], Kurdish [10], and Italian [11]); Ivs2 + 1G > A (Algerian [3]); Cys43Stop (Turkish [18]); Arg71Pro (Dutch [17]); Cys77Ala (Tunisian [4]); Pro82Ser (Turkish [19]); Gly86Arg (Palestinian [15], Turkish [15]), and Pakistani [16]), Korean [20], Taiwanese [21]); Arg96Stop (Croatian [9], Turkish [10], Korean [20], and Pakistani [16]); Lys98Pro (Turkish [22]); Cys99Tyr (Tunisian [12]); and Arg71His (patient was reported in a study from France, and the origin is not reported [23]).

The high consanguinity rates, coupled to the large family size in some communities, could induce the expression of autosomal recessive diseases, including new or very rare disorders such as MDM. Limited number of MDM cases was reported in several parts of the world [3, 4, 9–11]. The major reported cases of MDM in North Africa are of Tunisian descent with a total of 49 patients including the patients reported herein [3, 4, 12]. MDM is caused by mutations in *SLURP-1* gene encoding for SLURP-1 protein. SLURP-1 consisted of 103 amino acids and five disulfide bridges that are critical for the correct folding and function of the protein. SLURP1 potentiates the action of acetylcholine on the *α*7 nicotinic receptor [13], which plays an important role in the differentiation of stratified squamous epithelium [6, 14]. To date, only fourteen *SLURP-1* mutations were described in relation with MDM phenotype. Three of them are reported in Tunisia: C77A, 82delT, and p.Cys99Tyr (Figure 3).

In this report, we further characterize new MDM cases from Tunisia. Three families were recruited with variable clinical features of MDM. We found the 82delT variation commonly in families MDM-12 and MDM-13. This frame shift mutation results in truncated, misfolded, and therefore nonfunctional protein. The 82delT variation was previously identified across several ethnicities (Tunisian, Scottish, Algerian, Croatian, Kurdish, and Italian). It seems to be specific to the Mediterranean region (including 7 Tunisian and 10 Algerian families) suggesting the existence of a founder effect likely of Mediterranean origin where intermarriage is common [24].

The p.Cys99Tyr variation was identified in family MDM-14 that originated from the South of Tunisia. It affects a cysteine implicated in one of the highly conserved disulfide bridges. Although p.Cys99Tyr is towards the end of the protein, 3D folding showed that this mutation which is within loop3 of SLURP-1 caused difference in the 3D structure compared to the wild-type SLURP-1. It was suggested that mutations involving amino acids in loop3 might affect the binding of SLURP-1 to *α*7-nAchR [23]. The p.Cys99Tyr variation was previously identified only in Tunisian families [3, 4]. This sharp geographical demarcation is suggestive of a founder effect of Tunisian origin. Nevertheless, since some populations like Libya, Morocco, and Egypt have not yet been investigated, a more extended geographical distribution of this mutation could not be ruled out. 

Comparing the disease severity between the studied families, we observed that the clinical picture varied from severe to milder transparent hyperkeratosis. The most severe phenotype was observed in family MDM-12; however, the two other patients presented a moderate phenotype. In family MDM-14, multiple keratolytic pits were observed in the hyperkeratotic area which was not previously reported. Although these unusual keratolytic pits appeared in early infancy in the palmoplantar area, the phenotype in both affected patients is currently moderate but might exacerbate with age and exposure to mechanical or heat trauma.

In the literature, The extensive scarring on the hands and feet could be associated with melanoma development. Three cases of malignant melanoma arising in the hyperkeratotic lesions of MDM have been described in the literature at late age [21, 25, 26]. One case with classical clinical MDM finding complicated by irregular hyperpigmented spots on the palmoplantar regions and the back of hands and feet was also reported [27]. We think that unfavorable environmental factors and either bacterial or fungal infection play a major role in the worsening of MDM and may increase the skin thickening. No melanoma symptoms were observed so far in our cases even in family MDM-12 presenting the most pronounced MDM phenotype, but it could occur at late age.

## 4. Conclusion

In this report, we further characterize new MDM cases and identified the responsible mutations. In Tunisia, the MDM patients become aware of the risk to transmit the condition to the offspring. Therefore, we believe that it is very important to inform affected families about heterozygous carriers to avoid other consanguineous marriages. While the mutational findings have improved the understanding of MDM phenotype, it is necessary to continue with therapeutic assays of SLURP-1. The function of this protein needs to be further elucidated, and therapeutic approaches for this palmoplantar keratoderma are highly required. 

## Figures and Tables

**Table 1:** Pedigrees of the families and sequence electropherograms.

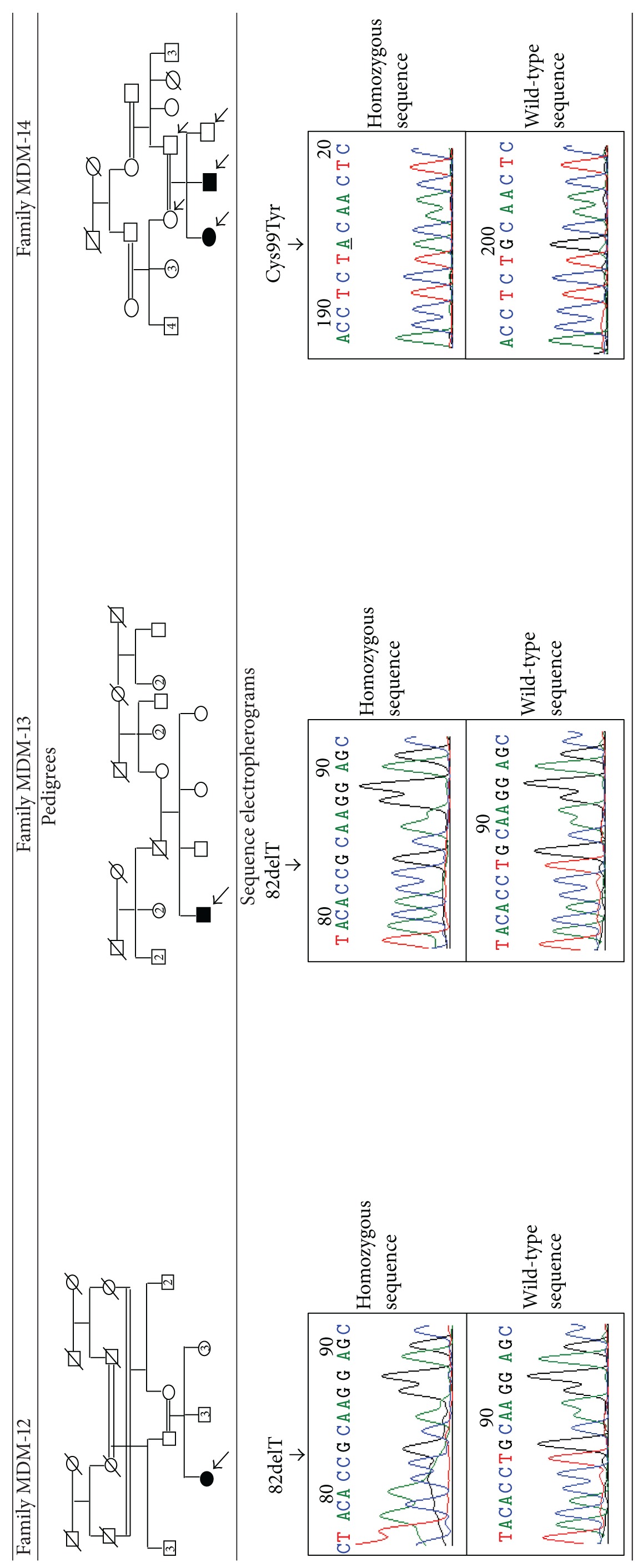

**Table 2:** Clinical characteristics of the Mal de Meleda patients.

Patient's characteristics	Patient in family MDM-12	Patient in family MDM-13	Patients in family MDM-14	
			Patient 1	Patient 2
Age (years)	40	40	2	13
Age of onset	First months	First months	First months	First months
Sex	F	M	M	F
Diffuse hyperkeratosis	++	++	+	+
Characteristics of transgressiveness	+++	++	+	+
Elbows involvement	++	−	−	−
Knees involvement	++	−	−	−
Palmoplantar hyperhidrosis	++	+	−	−
Malodor pachyderma	+++	++	+	+
Nails involvement	+++	++	++	++
Perioral erythema	+++	+	−	−
Keratolytic pits	−	−	++	++
Melanoma symptoms	−	−	−	−
